# Fluorinated Agents Effects on Orthodontic Alloys: A Descriptive In Vitro Study

**DOI:** 10.3390/ma15134612

**Published:** 2022-06-30

**Authors:** Roberta Condò, Elisabetta Carli, Alessandro Cioffi, Maria Elena Cataldi, Vincenzo Quinzi, Adriano Casaglia, Aldo Giancotti, Paola Pirelli, Ivano Lucarini, Francesco Maita, Luca Maiolo, Gianluca Mampieri

**Affiliations:** 1 Department of Clinical Sciences and Translational Medicine, University of Rome “Tor Vergata”, Via Montpellier 1, 00133 Rome, Italy; cioffi@amm.uniroma2.it (A.C.); melena.88@hotmail.it (M.E.C.); adriano.casaglia@gmail.com (A.C.); giancotti@uniroma2.it (A.G.); p.pirelli@gmail.com (P.P.); gianluca.mampieri@uniroma2.it (G.M.); 2 Department of Surgical Medical and Molecular Pathology and Critical Care Medicine, University of Pisa, 56126 Pisa, Italy; elisabetta.carli@for.unipi.it; 3 Department of Life, Health & Environmental Sciences, Postgraduate School of Orthodontics, University of L’Aquila, 67100 L’Aquila, Italy; vincenzo.quinzi@univaq.it; 4 Institute for Microelectronics and Microsystems—Unit of Rome, National Research Council, Via del Fosso del Cavaliere 100, 00133 Rome, Italy; ivano.lucarini@gmail.com (I.L.); francesco.maita@cnr.it (F.M.); luca.maiolo@cnr.it (L.M.)

**Keywords:** corrosion, orthodontic alloy, fluorinated mouthwash and gel, weight loss, scanning electron microscope, mass spectrometry

## Abstract

Fluoride-based mouthwashes and gels are preventive measures in countering demineralization and caries but, modifying environmental acidity, can reduce the wet corrosion resistance of orthodontic alloys. To evaluate chemical stability, in vitro experiments were conducted on stainless steel and nickel–titanium wires, weighed before and after immersion in household fluorinated mouthwashes and gels, measuring weight variations and elution of metal ions from acid corrosion phenomena. Elution samples were analyzed by inductively coupled plasma mass spectrometry, detecting residual ion concentration, while surface changes were analyzed under scanning electron microscopy. Results showed stainless steel wires do not undergo significant erosion when exposed to most fluorinated mouthwashes but, at prolonged exposure, alloys elute gradually greater amounts of metals and Ni–Ti wires become more sensitive to some mouthwashes. Ions’ elution varies considerably, especially for Ni–Ti wires, if exposed to household fluorinated gels, for which significant negative values were obtained. Changes, affecting wires’ outer layer, negatively act on shiny appearance and luster, reducing corrosion resistance. Although examined orthodontic wires showed good chemical stability and low toxicity, surface corrosion from exposure to fluorinated agents was observed. Home use must be accompanied by clinician prescription and, for household dental gels, must follow manufacturers’ recommendations, ensuring prophylactic action without damaging alloys surfaces.

## 1. Introduction

Although fixed orthodontic appliances principally consist of metals, materials considered non-cytotoxic in most conditions, during clinical use alterations may occur, thus changing their chemical properties which can lead to the manifestation of adverse effects, including the release of constituent elements such as ions from alloys [1]. The biodegradation of metal orthodontic appliances is a well-known adverse phenomenon that is first and foremost the primary cause of local and systemic reactions of hypersensitivity, due precisely to the release of certain metal ions such as nickel (Ni) and chromium (Cr) [2]. It has been observed that the metal ions released can be incorporated into the hard dental structures, staining them or even causing structural or functional damage to the appliance. For this reason, a primary requirement of any metal alloy is the ability to avoid the production of corrosion products which can be harmful to the body [3].

However, it has been shown that there is a continuous reaction of orthodontic wires and brackets with the hostile environment of the oral cavity, in which metal components are continually released. It has been also demonstrated how alloy composition can influence the corrosion resistance phenomenon [4]. Indeed, several conditions, including masticatory forces, orthodontic loads, fluctuations in temperature and pH, contribute to triggering these corrosion phenomena. Additionally, the electrochemical processes play a crucial role in corrosion when there are two alloys and a medium such as electrolyte. Indeed, the alloy with lower corrosion resistance acts as an anode and dissolves in the electrolyte and the ions are then released. On the surface of orthodontic wires, electrochemical corrosion, or wet corrosion, can therefore occur, even in the presence of a fluid electrolyte such as saliva, and this determines the elution of metal ions or the formation of chemical compounds produced by electrochemical reactions to which orthodontic alloys have been submitted [5]. Orthodontic alloys used for the manufacture of arches must be highly reactive base metals, with good biocompatibility and a high resistance to corrosion. This latter property is ensured by the presence of chromium in a metal mixture, a greatly reactive base metal that is able to spontaneously form a passive and protective surface film [6,7]. Oxygen is necessary for the forming and maintaining of the the film, while some microorganisms, such as *Bacteroides corrodens* and *Streptococcus mutans*, can also cause degradation of metal orthodontic alloys during long-term exposure [8]. 

During the fixed orthodontic treatment, in addition to periodontal implications phenomena of demineralization of the dental enamel may frequently appear, such as white spots or even caries, for this reason, the regular use of oral products containing fluorides is essential since the fluoride ion, favoring the formation of calcium fluoride globules, can promote tooth remineralization process [9]. Oral care products containing fluoride have a variable concentration of fluoride ions (250–10,000 mg/L) with pH values ranging from 3.5 to 7. Fluoride-based gels with a low pH have proven to be more effective in increasing the formation of calcium fluoride (CaF_2_) [10]. Some studies have investigated how fluorinated agents, depending on their acidity, are able to modify some of the main mechanical properties of orthodontic alloys. The effects of fluoride solutions with different acidities on load-deflection characteristics of nickel–titanium (Ni–Ti) orthodontic wires have been in vitro investigated and it was demonstrated that the daily mouthwash with a fluoride solution with more acidic pH of 4 affected the Ni–Ti wires load-deflection characteristics during the unloading phase [11]. Lee et al. verified in vitro that Ni–Ti wires have dissimilar corrosion resistance in an oral environment containing fluoride, and this resistance does not correspond to the appreciated variations in the surface topography of the archwires. The presence of fluorine to 0.5% concentration of sodium fluoride (NaF) in the artificial saliva is normally detrimental to the corrosion resistance offered from these alloys [12]. The fluoride ions are very aggressive on the protective film of titanium dioxide present on the Ni–Ti wires and can cause greater corrosion of the orthodontic wire. Furthermore, depending on the concentration, the fluoride ions are able to penetrate the narrow gaps between the orthodontic wire and orthodontic bracket by attacking the interface. This in turn leads to an increase in surface roughness as well as greater friction [13,14]. The amount of ions released by Ni–Ti wires is always higher than that released by stainless steel (SS) wires and the release of ions from orthodontic wires in Ni–Ti increases with the pH decrease of the immersion solution [15,16].

Generally, studies focusing on ion release from orthodontic wires consist of exposing wire segments to a solution for a specified period of time. Often, the exposure solution is an artificial saliva solution. However, as there are many different compositions of artificial saliva, comparisons between these studies are problematic [17]. In reality, the test procedures for studying the biodegradation of metals, with methods that use non-biological systems, can be carried out, both through the use of inorganic-organic means, such as artificial saliva, but also with purely inorganic solutions such as the isotonic solution of NaCl. In fact, given their sensitivity in these investigations, both electrochemical methods are often used in gravimetric procedures, also allowing the determination of parameters such as pitting corrosion potential [18]. It has also been shown that the dissolution of alloys can be different depending on the experimental conditions, including the medium used which, based on its chemical composition and pH, is able to modify the speed with which the different metal ions are released from the alloy, considering also that some of them, including nickel, dissolve after a defined period under identical experimental conditions. Finally, it is generally recognized that nature, rather than the amount of corrosion products released, is important for cytocompatibility [17].

For these reasons, in the present in vitro study, normal saline was used as a control medium, a solution universally known for its composition (0.9% sodium chloride solution) and pH (5.5), which are important variables because of their constancy and ability to ensure the repeatability of the experimental method. In the literature, it is not surprising that the quantity of nickel released in general by alloy orthodontic appliances can also be influenced solely by the type of drink ingested [17].

The null hypothesis of the present in vitro study has assumed that the corrosion phenomena that occur following the immersion of orthodontic alloys in fluoride solutions are no greater than those that happen using a normal saline solution as eluent. The aims of the study were: (1) to evaluate, quantify and describe the release of metal ions from SS and Ni–Ti orthodontic wires after their exposure, in vitro, to other relevant specific media, such as fluoride solutions. These included five topical mouthwashes and two gels for home use, based on different fluoride agents, sodium fluoride (NaF) or amine fluoride (AmF) with specific concentrations of fluoride and pH, at predetermined time intervals; and (2) to assess the measurements of weight variations, obtained through these exposure protocols to understand a larger scale potential harmful corrosion phenomena.

This study intends to raise awareness among manufactures and practitioners of the correct home use of the caries preventive devices, both mouthwashes then fluorinate gels in particular, since these products can be accessed directly and indiscriminately by patients, without any medical prescription. It is essential to inform the patients about the possible interactions of these products with the orthodontic alloys, to avoid changes in the mechanical properties of the appliances, or worse, the ingestion of eluted metal particles from damaged archwires during the treatment.

## 2. Materials and Methods

### 2.1. Sample Preparation Protocol

Using a distal end cut plier in SS (Leone Spa, Sesto Fiorentino, FI, Italy), a total of 32 (*n* = 8) preformed rectangular orthodontic archwires: SS AISI 304 Extra-hard (C3112-17, Leone Spa, Italy) (1), SS AISI 302 Twist (C5745-17, Leone Spa, Italy) (2), Ni–Ti (C5912-17, Leone Spa, Italy) (3) and SS AISI 304 (S3TFL1725, G&H Wire Company, Franklin, IN, USA) (4), with equal dimension of 0.017 × 0.025 inch, have been manually sectioned in three parts of 3 cm in length each, by the same operator, thus obtaining 24 sections of each type of alloy for a total of 96 (*n* = 24) arch section samples. All sections of wire were cleaned using acetone solution, rinsed with distilled water and dried [19].

At first (t_0_), sections have been weighed (w_0_) using a precision balance Mettler, Toledo XS204 scales (ME104TE/00, Cole-Parmer, IL, USA) with 0.1 mg accuracy and then placed into a sterile 15 mL Falcon tube (339650, Thermo Fisher Scientific, Cambridge, UK). 

Each tube was filled with 10 mL of only one of the six topical fluoride mouthwashes or only one of the two gels, in order to completely immerse each section and, a control group consisting of normal saline was added to the study protocol (Table 1).

The specimens, until the in vitro tests, were stored at a controlled temperature of 37 °C, for three different time storage intervals: 1 (t_1_), 24 (t_2_), and 168 (t_3_) hours. 

At the end of each established storage time, each section was extracted from the test tube, immersed in deionized water for 30 min and then dried with a jet of pure nitrogen and weighed (w_1_, w_2_, w_3_, w_4_, w_5_) for five times always by the same operator. 

Each eluate, residual from the immersion of the arch sections at predetermined time intervals, was analyzed by mass spectrometry (ICP-MS of AGILENT Mod. 7800) (Agilent Technologies, Tokyo, Japan) in order to search for the possible presence of metal ions, such as Ni, Cr, and Iron (Fe) resulting from the possible acid corrosion of surfaces exposed to the fluorinated agents contained in mouthwashes and gels.

The analysis was carried out by diluting the liquid samples of mouthwash in acidified water, with ultrapure nitric acid, and then sonicated for 30 s. The gel samples, on the other hand, were weighed (w_1_, w_2_, w_3_, w_4_, w_5_) five times, always by the same operator, mineralized in a microwave mineralizer with the addition of ultra-pure nitric acid and reported to a known volume.

Blank samples (Bs) of the eight pure products, or reagents, analyzed, were also realized.

The four different archwires have been cut into 2 cm long pieces by a distal end cut plier in SS (Leone Spa, Sesto Fiorentino, FI, Italy), washed in isopropyl alcohol and rinsed in deionized water. Then the four samples were dried using a nitrogen flux and loaded into a Gemini1 Zeiss SEM to be analyzed in detail.

All samples, including Bs, were subsequently analyzed using an inductively coupled plasma mass spectrometer (Agilent 7500c ICP-MS, Agilent Technologies, Tokyo, Japan) equipped with an integrated auto-sampler (Agilent Technologies, Tokyo, Japan) and an Octupole reaction system and ICP torch, consisting of a three-cylinder unit, with a 2.5 mm diameter injector. This sensitive instrumental technique is able to determine the presence of various metallic and non-metallic inorganic substances, present in concentrations of approximately one part per billion (ppb). By means of a Babington PEEK (poly-ether-ether-ketone) nebulizer matched with a double-pass spray chamber (Agilent Technologies, Tokyo, Japan), and the samples were inserted. To guarantee the stability of the temperature and minimize the water vapor in the sprayer gas flow, the spray chamber was water-cooled to 2 °C. The different metal ions constituting the alloys of the different orthodontic wires under examination were measured and, in particular, Cr^52^, Fe^56^, and Ni^60^ were determined in all the experimental groups, regardless of the alloy compositions supplied by the manufacturers. Three determinations were then obtained for each sample, therefore, the result of a sample is the average of the three values. The metal concentration is on the order of one part per billion, equivalent to 1 mg per liter.

### 2.2. Statistical Analysis

One-way ANOVA for each individual substance in which the four different orthodontic alloys were immersed for three different time intervals was performed. 

The weighted average of the five weighing was calculated and finally the relative difference from the initial weight was obtained. 

None of the variations of 0.4 and 0.5% were considered as significant as they were within the measurement error that was calculated for each measured for both positive (increasing) and negative (loss) values. Negative values higher than 1% are instead considered significant because they all represent real losses not dependent on the measurement procedure.

As regards mass spectrometry, from each group ten samples were measured and averaged to obtain the final ion concentration. Each measurement was collected with an integration time of 0.5 s.

## 3. Results

### 3.1. Weight Loss Tests

**Figure 1:**
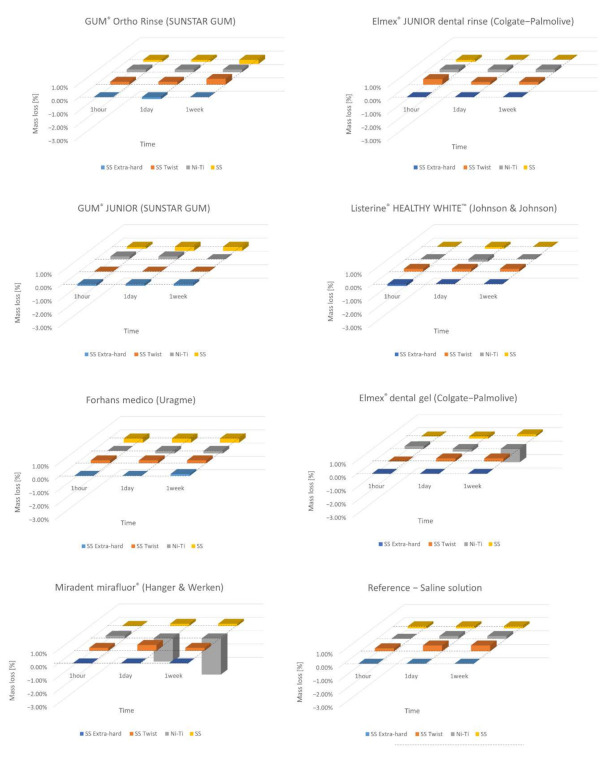
Differences in weight percentage of the 96 orthodontic arch section samples (*n* = 24) after 1 h (t_1_), 24 h (t_2_) and 168 h (t_3_) of storage in the different fluorinated agents. One-way ANOVA for each individual substance in which the four different orthodontic alloys were immersed for three different time intervals has been performed. The weighted average of the five weighings was calculated and finally the relative difference from the initial weight was obtained.

In Figure 1 are shown the values, expressed in difference of weight percentage, of the final weighs (w_1_, w_2_, w_3_) of all the specimens under examination. The specimens have been maintained in the specific solution and, at fixed times, they have been dried and weighed. The average values have been used as reference data for the calculations of the weight lost percentage at different times (t_1_, t_2_, t_3_). Weight variations corresponding to the systematic error of the precision balance (±50 µg) are not considered significant. Additionally, variations above or below 1% are not considered significant. In all alloys, at different time points, after immersion in the fluorinated agents but also in saline solution, some slight increases in weight were recorded.

The SS AISI 302 Twist wire samples (2), among all, show the best dimensional stability for all fluorinated solutions at 1 (t_1_) and 24 (t_2_) hours, although they have the largest exposed surface since it is made up of eight thin rectangular wires intertwined with each other. Samples in SS AISI 304 Extra-hard (1) were found to be less resistant to corrosion than SS AISI 304 (4) wires, after immersion at 1 h (t_1_) and 24 (t_2_) hours, despite the alloy with which they are both made having the same AISI classification, while at 1 week their behavior looks similar. 

Ni–Ti (3) samples, after an initial weight increase at 1 h of immersion (t_1_), present a certain stability even after a week of immersion (t_3_) in AmF (0.025%) and NaF (0.02%) mouthwashes. 

A significant weight loss has been observed only for prolonged immersion times (t_3_) in fluorinated gels, especially for Ni–Ti orthodontic arches.

### 3.2. Scanning Electron Microscope Characterizations

**Figure 2:**
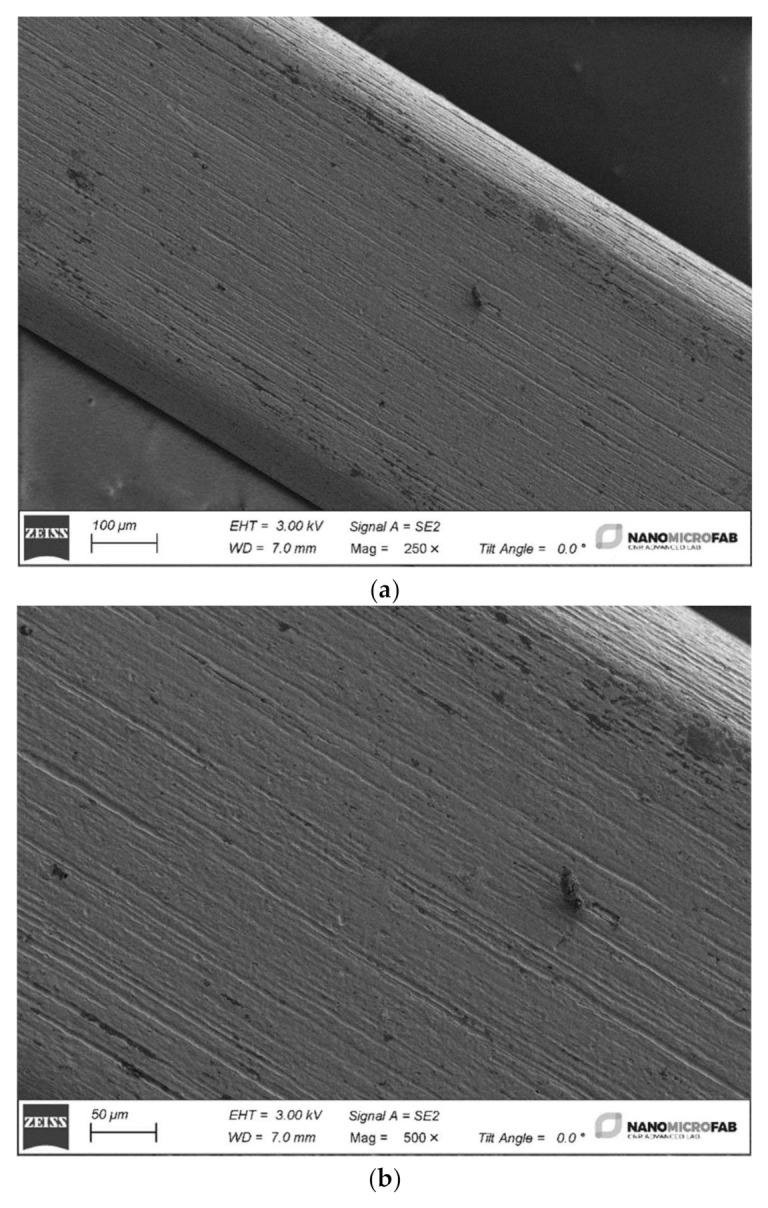

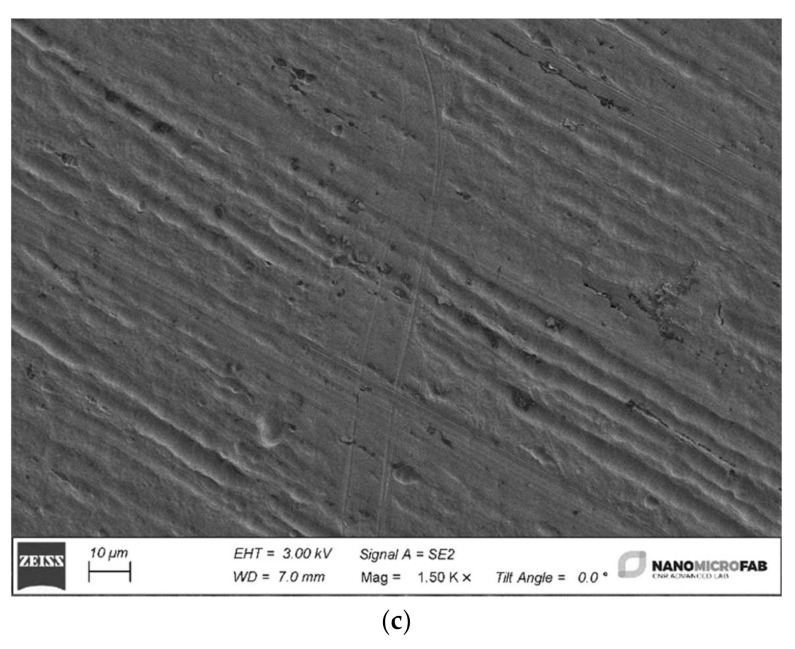
Three images of a Ni–Ti archwire sample captured at increasing magnification: (**a**) image at 250×; (**b**) image at 500×; (**c**) image at 1500×.

**Figure 3:**
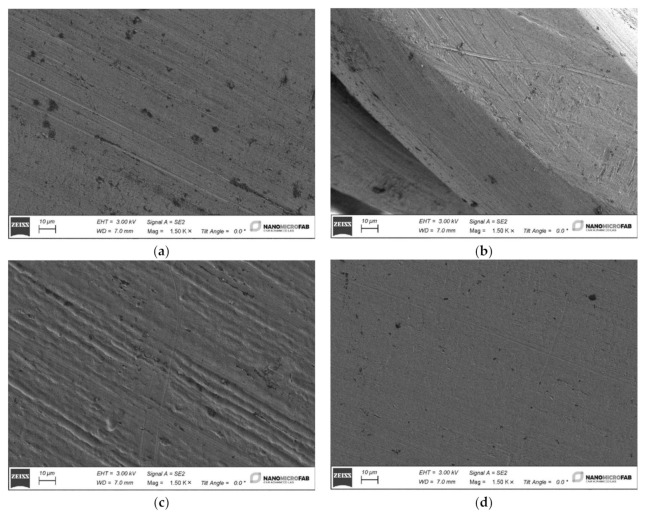
Four SEM images of the different “as is” samples at a magnification of 1500×: (**a**) SS AISI 304 Extra-hard; (**b**) SS AISI 302 Twist; (**c**) Ni–Ti; (**d**) SS AISI 304 S.

In Figure 2, the images of Ni–Ti archwire (3) surface at increasing magnification is observed while, in the Figure 3, the surfaces of the four samples, before the ageing in the proposed fluorinated products (“as is” samples), are presented. 

Despite cleaning, the surface has some dirt that is possible to observe as black dots. Both the archwires made from stainless steel or Ni–Ti reveal a longitudinal series of grooves produced by the mechanical manufacturing process.

After the aging in the different mouthwashes and dental gels, in all the samples, a significant improvement in the cleaning of the surface can be appreciated. 

**Figure 4:**
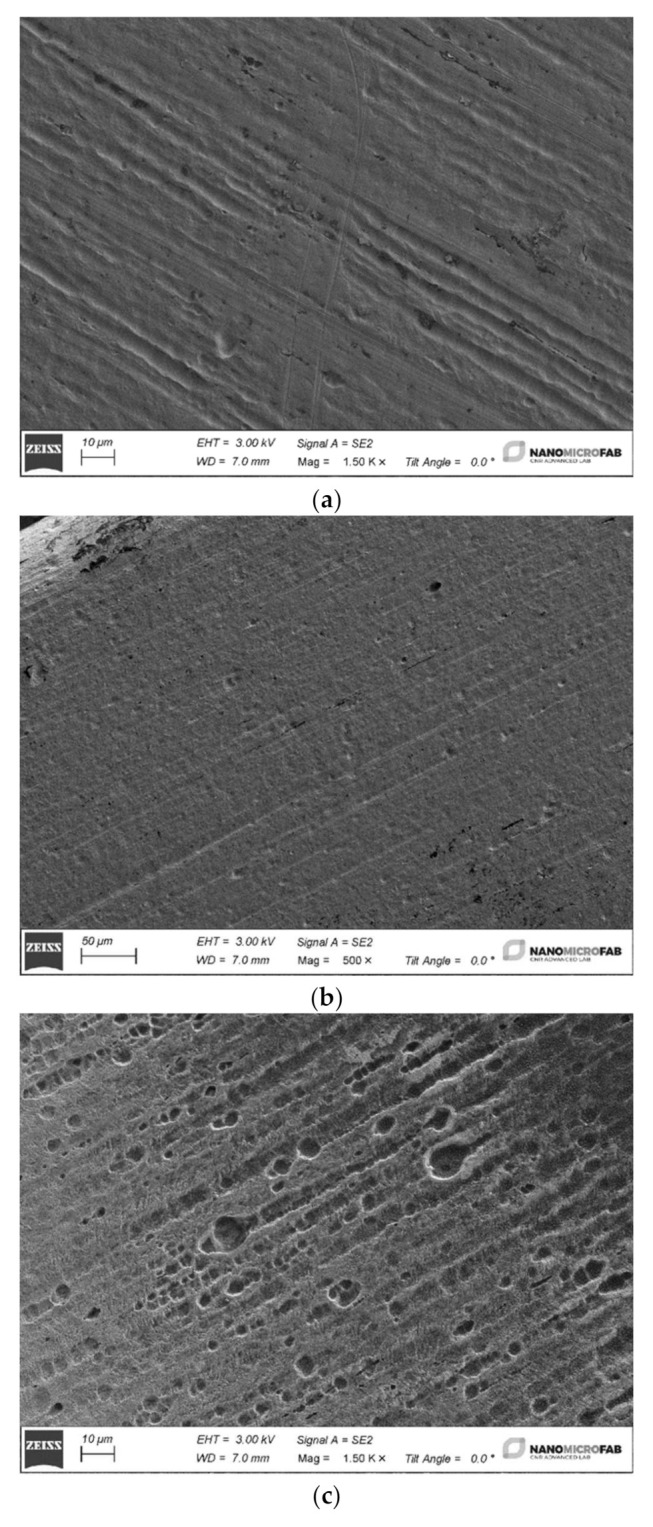
(**a**) An image of the Ni–Ti sample before the ageing test; (**b**) an image of a Ni–Ti sample at lower magnification after one week of aging in Miradent mirafluor^®^ dental gel; (**c**) the surface of the Ni–Ti sample after the ageing for one week in Elmex^®^ dental gel.

Especially after one week, almost all the impurities have been removed, as shown in Figure 4a,b. Additionally, the one-week aging in dental gel provoked a visible, superficial corrosion in samples made of Ni–Ti alloy.

The presence of small holes that are uniformly distributed along the archwire surface (Figure 4b) can be observed. 

These damaging effects are also confirmed in the weight loss and mass spectrometry tests results.

### 3.3. Mass Spectrometry Tests

The collected data on the concentration of Cr, Fe, and Ni ions present in the eluates, resituated from the immersion of arch sections in the different solutions at predetermined time intervals, are shown in Table 2. 

In normal saline solution, at 1 h (t_1_) of immersion there is no release of Cr from the alloys examined, it is present in minimal quantities only in the residual eluate after 24 h (t_2_) of immersion of the SS AISI 304 (4) wires, but at 168 h (t_3_) Cr is released in minimal quantities (9.27 µg/kg) from all orthodontic archwires, in particular from SS AISI 302 Twist (2) wires. In normal saline solution, on the other hand, Fe ion is always present: from the first hour (t_1_) of immersion it is released from all alloys (+/−10 µg/kg), at 24 h (t_2_) its value tends to triple (33.2 µg/kg) for SS AISI 304 (4) wires and at 168 h (t_3_) it increases (69.4 µg/kg) for all orthodontic wires, especially for SS AISI 302 Twist (2) in which the eight thin metallic wires, intertwined with each other, offer a greater exposed surface. Ni, on the other hand, is not released by SS AISI 304 Extra-hard (1) and SS AISI 304 (4) wires at no immersion time, in the orthodontic archwires SS AISI 302 Twist (2) slightly increases from t_1_ to t_2_ and then its value quadruples to t_3_ while in the wires 0.017 “× 0.025” Ni–Ti (3) increases, doubling from t_1_ to t_2_ and multiplying nine times from t_2_ to t_3_.

In the different fluorinated mouthwashes, at 1 h (t_1_) of exposure, Cr is not present in the various eluates in a very significant way, in fact its concentration appears almost superimposable to the Blank samples (B); a maximum value (40.8 µg/kg) is recorded for it only at 168 h (t_3_) of immersion of the threads SS AISI 302 Twist (2) in NaF (0.02%) (E) mouthwash. Therefore, in the different fluorinated mouthwashes, as the immersion time increases, orthodontic alloys elute gradually greater quantities of metal ions. This general increase is recorded to be more constant and linear for the Cr ion and more variable and inconstant for both Fe and Ni ions which, at 168 h (t_3_), seem to show the most significant increases. In general, Cr is the ion that was most released by orthodontic arches SS AISI 302 Twist (2) in contact with all the mouthwashes under examination for all the times considered. Fe is eluted in greater quantities than chromium and mainly from wire SS AISI 304 Extra-hard (1) after immersion in mouthwashes based on NaF 0.04% (A), NaF 0.0226% (C) and NaF 0.022% (D) and orthodontic archwires SS AISI 302 Twist (2) exposed to fluorinated agents AmF 0.025% (B), NaF 0.0226% (C) and NaF 0.022% (D). Fe ion, on the other hand, is almost always present in eluates in greater quantities than chromium. These values, in fact, are more significant when the orthodontic alloys are exposed to the mouthwash based on NaF 0.022% (D), while with that based on NaF 0.02% (E) at 168 h (t_3_) it undergoes an abrupt halving. Finally, Ni is present in general in lower quantities than Fe ion and is always released by the alloys of Ni–Ti (3) wires exposed to all fluorinated mouthwashes and especially those based on AmF 0.025% (B) and NaF 0.02% (E) at the maximum exposure times. Immersion of the orthodontic alloys in fluoride gels revealed that, at both t_1_ and t_2_, Cr and Fe ions are released in a similar manner, in particular Cr is further eluted following exposure to AmF (1.25%) (F) while at t_3_ it was eluted more by coupling the NaF gel (1.23%) (G) with SS AISI 302 Twist wire (2), while Ni ions are released in general much more and, in particular by the Ni–Ti wires when exposed to the two fluorinated gels, for which sensitive negative values have been obtained, i.e., above 1% until reaching for the gel G maximum loss of 2.7% at 1 week of exposure.

## 4. Discussion

Fluoride-based mouthwashes and gels are very effective preventive oral devices to counteract the onset of dental caries and for this reason they have always been widely used in fixed orthodontics. On the other hand, it has also been observed that they are able to modify the environmental acidity, thus reducing the capacity of resistance to wet corrosion offered by pure titanium and its alloys due to the breaking of the protective layers of surface oxide and thereby corroding stainless steel dental appliances. It is known that the corrosion resistance of titanium is strongly dependent on the concentration and pH value of the fluoride contained in the various formulations for home and professional topical use [20].

Corrosion phenomena of orthodontic wires are able to release heavy metals into the oral cavity such as Cobalt, Cr, Ni and other ions [21]. 

In austenitic steels that contain Ni as a primary austenite stabilizer, Ni atoms are not strongly bonded to form an intermetallic compound so the likelihood of slow release of Ni ions, in vivo, from the alloy surface is higher, which can have biocompatibility consequences [22,23,24,25]. 

In vivo, it was demonstrated that Ni concentrations in saliva increased after the placement of SS bands and brackets but decreased to the starting levels two weeks after the placement of appliances. Subsequently, the placement of a Ni–Ti wire also determines an elongation of this effect which, however, decreases within 10 weeks [26].

Ni is the most common cause of metal-induced allergic contact dermatitis in humans and produces more allergic reactions than all other metals combined. Cr is second in frequency. In fact, extra oral adverse reactions are much more common than intraoral ones and allergic skin reactions are also difficult to distinguish from irritative lesions [27].

There is evidence in the scientific literature to support the fact that Ni has carcinogenic, mutagenic, and cytotoxic effects in cell cultures [1,22,23,27]. These findings should be interpreted with caution, because documented toxicities generally apply to the soluble forms of these elements. Currently, any association between the release of metal and metabolic, immunologic, or carcinogenic toxicity is conjectural because a cause-and-effect relationship has not been demonstrated in humans.

In an in vitro immersion study performed in 2004 by Eliades et al., ions released from both SS and Ni–Ti brackets and wires did not report any measurable effect on the vitality and physiology of the periodontal ligament and gingival fibroblasts [22]. 

However, studies have investigated the release of metal ions from orthodontic alloys following contact, more or less prolonged, with fluorinated agents. In environments containing fluoride dissociated into ions, a reduction in corrosion resistance of pure titanium and titanium alloys has been demonstrated [27,28,29].

Additionally, the exposure of Ni–Ti orthodontic wires to NaF-acidulated topical products causes a production of fluoridric acid, which quickly dissolves the titanium, resulting in the corrosion of the metal alloy [30].

In 2002, Schiff et al. compared the electrochemical properties of different titanium alloys with respect to fluoride ion content and salivary pH. Although Ni–Ti and Ni–Ti–Co were found to be less affected by corrosion phenomena, fluoride ions had negative effects on all materials [31]. Furthermore, in 2004 Schiff et al., measuring corrosion related to the use of fluorinated mouthwashes on titanium orthodontic alloys, observed that NiTi-based alloys are strongly corroded in the presence of monofluorophosphate, TMA wires are intensely corroded with stannous fluoride, while the TiNb archwires demonstrate the greatest resistance to corrosion. According to the authors, it is therefore necessary, based on the orthodontic alloy used, to advise the patient on which is the most suitable fluorinated agent to perform oral rinses [10].

As regards weight loss tests performed in the present in vitro study, minimal changes were recorded, of the order of 1%, in defect or in excess for all the stain steel samples. We did not consider these variations significant, according to the specific measurement procedure. For the Ni–Ti arches, we observed higher weight loss in the case of gels, especially after 1 week (t_3_) of immersion (samples F3 and G3) (Table 2). Considering these observations, all the orthodontic materials reveal a good chemical stability to corrosion. In fact, we can estimate the immersion time of 1 h (t_1_) of the metal sections to be approximately two months of oral exposure of an orthodontic wire during a fixed orthodontic treatment, for a patient who consistently performs two rinses lasting 30 s each with a fluoridated mouthwash. Multiplying these by 30 days, it results that in one month, 1800 s, that is, 30 min are spent rinsing with fluoride mouthwash. This means that in 2 months, 3600 s, or 60 min and then 1 h (t_1_) is spent performing rinses with a fluoride mouthwash. Moreover, a significant weight loss has been observed only for prolonged immersion times (t_3_) in fluorinated gels, especially for Ni–Ti orthodontic arches.

These findings can be confirmed by mass spectroscopy tests (Table 2), where consistent amount of metal ions (Ni) have been collected in the samples F3 and G3. 

The tests carried out on the weight loss of orthodontic archwires exposed to fluorinated agents with different concentrations of fluorine have shown minimal and not significant weight variations. These changes, even if marginal, have affected the external layer of the wires and have negatively influenced the shiny appearance and the metallic luster of their surface. 

In all alloys, at different time points, after immersion in the fluorinated agents but also in saline solution, some slight increases in weight were recorded. This phenomenon is predictable and is linked to the cleaning phase of the sample previously exposed to fluorinated agent which is performed before the new weighing: the wire section is in fact immersed for 30 min in deionized water and then dried. This concurrent process, which can occur when these measurements are carried out, increases the initial weight due to further deposits of salts from mouthwashes and gels or even of organic material from gels which, according to the nature and composition of the fluorinated solution (gels are more viscous), can chemically adhere to the most superficial layer of the metal alloy section, effectively increasing its weight.

From the results obtained from the mass spectrometry tests (Table 2) it emerged that even in normal saline solution the alloys seem to show a neutral or inert behavior, they do not seem at all free from corrosion, and this allows acceptance of the null hypothesis of the present in vitro study. These phenomena can be explained by the presence in the saline solution of components that can form chloridric acids acting as an etching agent for the alloy.

There are many published studies on the corrosion resistance of Ni–Ti alloys in physiological solutions [8,21,27,28].

Considering exposure to different fluorinated mouthwashes, it is observed that in general, as the immersion time increases, orthodontic alloys elute gradually greater quantities of metal ions. This general increase is recorded to be more constant and linear for the Cr ion and more variable and inconstant for both Fe and Ni ions which, at 168 h (t_3_), seem to show the most significant increases. In general, Cr is the ion that was most released by orthodontic wires SS AISI 302 Twist (2) in contact with all the mouthwashes under examination for all the times considered.

Fe ion, on the other hand, is almost always present in eluates in greater quantities than chromium. These values, in fact, are more significant when the orthodontic alloys are exposed to the mouthwash based on NaF 0.022% (D), while with that based on NaF 0.02% (E) at 168 h (t_3_) it undergoes an abrupt halving. 

Finally, Ni is the metal ion present in general in lower quantities than Fe ion and is always released by the alloys of Ni–Ti (3) wires exposed to all fluorinated mouthwashes, and especially those based on AmF 0.025% (B) and NaF 0.02% (E) at the maximum exposure times. Jamilian et al. showed that the amount of ions released by the Ni–Ti (3) wires is higher than that released by the steel wires and that this phenomenon is closely related to the increase in pH in the oral cavity [15]. 

The mouthwashes that prove to have caused a greater release of ions in general are those based on NaF 0.022% and NaF 0.02%. Cr was released to a similar extent especially after exposure to NaF 0.04% (A) and AmF 0.025% (B), Fe especially after immersion in NaF 0.04% (A) and equally from AmF 0.025% (B), while NaF 0.0226% (C) was the least aggressive also towards Ni. AmF 0.025% (B) and NaF 0.02% (E) show the greatest peak of increase after 1 week of contact thus demonstrating the greatest release of nickel from Ni–Ti wires at 1 week (t_3_). Barret et al. also evaluated the release of Cr and Ni from Ni–Ti and SS wires at 1, 7, 14, 21, and 28 days showing that Ni release reaches a maximum after approximately 1 week [32].

The data included in Table 2 allow us to state that the different fluoride agents have had a negative effect on all the orthodontic archwires under examination, causing, in general, a reduction in their ability to resist metallic corrosion as the exposure time increased. 

Corrosion is known to have significant consequences for the mechanical properties of orthodontic alloys, such as an increase in surface roughness and weakening of their resistance, which can lead to mechanical failure or even wire fracture [2]. On the other hand, it was also the cause of the release of variable quantities of metal ions from the surface layers of the arches in exam (Table 2). 

The elution of metal ions from the SS wires, in all the tested fluorinated mouthwashes and for all the time intervals considered, was found to be within the biological safety threshold, also with regard to the relative toxicity of Ni. Castro et al. reported that in SS alloys which have a Ni content of 8.00%, the crystal lattice binds the nickel ions, making them unavailable to react. Therefore, these low Ni alloys are not capable of causing Ni hypersensitivity, being tolerated by Ni-sensitive patients [33].

In the safety data, tables relating to the composition of the archwires alloys in SS list the following values relating to Ni content: 8.00–10.50% for SS AISI 304 Extra-hard (1), 8.00–10.00% for SS AISI 302 (2), and 9.00% for SS AISI 304 (4). From Table 2 it can be seen that, as regards the release of Ni ions after immersion in all five fluorine mouthwashes, the three alloys in SS assume a rather similar behavior, which can be represented by the following equation:

A = C < E < D < B

where NaF (0.04%) (A) and NaF (0.0226%) (C) mouthwashes determine the lowest release of Ni, therefore among all mouthwashes they were found to be the least corrosive towards orthodontic alloys in SS. On the contrary, the AmF (0.025%) (B) mouthwash is the most aggressive towards SS alloys in exam.

Jamilian et al. demonstrated that the amount of ions released by the Ni–Ti wires is higher than that released by the SS wires and this phenomenon is closely related to an increase in oral pH [15]. 

As for the orthodontic wires in Ni–Ti (3), they have proven to be strongly corroded only if immersed in AmF (0.025%) and NaF (0.02%) mouthwashes at the maximum exposure times. The release of Ni ions, consequent to this corrosive phenomenon, was, however, significant since the concentration of Ni found in the eluates with the AmF (0.025%) (B) and NaF (0.02%) (E) mouthwashes was found to be greater than the biocompatibility threshold values. In fact, the daily intake of Ni from food and drinks is approximately 300–500 µg (Chromium is 5 to 100 µg per day) and it is confirmed that if this absorption exceeds 2.5 µg/kg, allergic symptoms may appear, as the concentrated dose required for allergic reactions is 600–2500 µg [34,35].

This data is to be considered significant only for long and continuous exposure times because we observed that the amount of released metal ions by Ni–Ti wires increased with immersion time, but it should also be considered that the length of an orthodontic archwire is generally greater than the 3 cm considered in this study. Therefore, to obtain metal sections useful for carrying out the investigation in vitro, the total release of Ni, as well as that of the other metal ions, is certainly greater and can be estimated at three to four times as much.

The results obtained after immersion of the wires in the fluoride gels have revealed that both at t_1_ and at t_2_ the Cr ion was further released following exposure to AmF (1.25%) (F) while at t_3_ was found to be further eluted by coupling the gel of NaF (1.23%) (G) with SS AISI 302 Twist (2) wire. With fluorinated gels, the Fe ion is eluted from the alloys in a very similar way to Cr, while Ni is released in general much more and in particular from the Ni–Ti wires after their exposure to the AmF gel (1.25%) (F) but above all to NaF (1.23%) (G), reaching toxicity threshold values even at intermediate immersion points. This phenomenon is certainly linked to the higher concentration of fluorides present in the formulations of dental gels.

In general, SS arches are more stable than those of Ni–Ti when immersed in fluorinated mouthwashes, except in gels. In fact, SS orthodontic wires do not undergo significant erosion when exposed to most of the fluorinated mouthwashes for home use, while it has been observed that, for prolonged exposure, Ni–Ti arches are more sensitive to some of them. This is also confirmed by the scientific literature. The elution of metal ions results to change significantly in terms of metal erosion especially for the Ni–Ti wires when exposed to the two fluorinated gels for home use, for which sensitive negative values have been obtained.

A recent study conducted in vitro on fixed orthodontic appliance exposed to soft drinks shows how Coca Cola^®^ causes an increase in the release of Ni, while orange juice does not intensify the release of metal ions so much [36]. Therefore, the acidity presence in the oral environment can be damaging for orthodontic alloys and, in reality, any substance with an acidic pH, when placed in contact with orthodontic metal alloys, can cause a dissolution of metal ions which will be greater the more acidic the pH of these solutions. This is why it is essential to faithfully respect the indications established by the manufacturer, both in terms of timing and methods of use, of these oral prophylaxis agents.

The regular use of fluoride-containing products during the course of orthodontic treatment is essential to promote and stimulate remineralization of tooth enamel. Fluoride mouthwashes, available in 0.05% and 0.2% fluoride concentrations, are often prescribed by orthodontists for weekly and even daily use to prevent dental caries [37]. 

Regarding professional topical application, compared to gels, fluorinated varnishes offer a definite advantage, represented by the possibility of selecting the site of clinical application. This prevents direct contact of the fluorinated agent with the alloys of arches and orthodontic brackets, thus avoiding all potential damages related to the acid dissolution of metal ions that occurs following the application of fluorides on these structures and in general, as happens when using fluoride dental gels [38]. 

In light of what emerged from this in vitro study, it is evident that, during fixed orthodontic therapy, the use of these effective anticaries devices must also take into account the risk factors related to the release of metal ions, such Fe, but above all Ni and Cr from the orthodontic wires and the phenomena of surface deterioration of the same, induced by corrosion from exposure to fluorinated agents. Since the products examined in this in vitro study are formulated for home use, being therefore public access, they do not require a medical prescription, not even for the examined gels that have the same concentrations of fluorides present in products for professional use. It is therefore essential to sensitize the orthodontic patient to their correct use, especially for home gels, strictly following the manufacturer recommendations. It could also be useful for the manufacturer to add a warning note in the product specifications that always refers to the opinion of the treating orthodontist, who can indicate to the patient the most suitable fluorinated agent for each sequence of orthodontic wires, thus avoiding exposing more sensitive wires to acid dissolution phenomena, such as Ni–Ti wires to fluorinated molecules with high acid-corrosive power, such as the gels examined. This would make it possible to really use these domestic devices in a preventive manner, guaranteeing a prophylactic action without damaging the surface structures of orthodontic alloys.

## 5. Conclusions

Although the initial hypothesis has been demonstrated, the results obtained in this in vitro study suggest that the concentrations of eluted metal ions are in any case negligible because they are minimal, not uniform in all the time intervals of the tests, and in any case more significant only at very long exposure intervals, the last condition being difficult to verify clinically. Therefore, even if the phenomena of corrosion of metals by fluorinated products exist, the orthodontic wires examined in this in vitro study showed good chemical stability and low toxicity. Acid pH substances, such as fluorinated agents and normal saline, cause dissolution of metal ions from orthodontic wires. The warning message concerns the two gels, which are in fact available as over-the-counter drugs and recommended for home use at concentrations of fluoride strictly similar to those intended for professional use. A fluorinated solution with a significant corrosive power, such as that demonstrated by fluorinated gels in exam, if arbitrarily used for incorrect times, is undoubtedly capable of damaging orthodontic alloys and in particular those of Ni–Ti, modifying their mechanical properties. For this reason, their home use should always be accompanied by careful prescription and supervision by the clinician in order to guarantee the prophylactic action without damaging the superficial structure of orthodontic alloys.

## Figures and Tables

**Table 1:** Fluoride mouthwashes, gels, and normal saline solution taken in exam in the in vitro study.

Group	Figure	Commercial Name	Fluorinated Agent	pH	Usage Recommendations
A	Mouthwash	GUM^®^ Ortho Rinse (SUNSTAR GUM)	NaF (0.04%)	6.5	rinsing with 10–15 mL for 30 s twice a day
B	Mouthwash	Elmex^®^ JUNIOR dental rinse (Colgate-Palmolive)	AmF (0.025%)	4.7	rinsing with 10 mL of product for 30 s
C	Mouthwash	GUM^®^ JUNIOR (SUNSTAR GUM)	NaF (0.0226%)	5.5	rinsing with 15 mL for 30 s twice a day
D	Mouthwash	Listerine^®^ Healthy White^™^ (Johnson & Johnson)	NaF (0.022%)	5.6	rinsing with 10 mL of product for 60 s
E	Mouthwash	Forhans medico (Uragme)	NaF (0.02%)	4.5	rinsing with 2 caps of product for 60 s twice a day, for at least 15 days
F	dental gel	Elmex^®^ dental gel (Colgate-Palmolive)	AmF (1.25%)	4.5	home application of 1 cm gel using the toothbrush for 2 to 3 min once a week
G	dental gel	Miradent mirafluor^®^ (Hanger & Werken)	NaF (1.23%)	5.1	home application of 1.5 cm gel using the toothbrush for 2 to 4 min once a week
H	normal saline solution	0.9% sodium chloride solution	-	5.5	-

**Table 2:** Concentration (µg/L) of metallic ions present in the eluates, resituated from the immersion of arch sections in the different solutions at predetermined time intervals. The cells showing the greatest release of ions were colored in green.

Concentration (µg/L) of Metallic Ions in Eluates at Predetermined Time Intervals													
Fluoride Mouthwashes, Gels and Normal Saline Solution	Orthodontic Archwires	Cr µg/L				Fe µg/L				Ni µg/L			
		Bs	t_1_	t_2_	t_3_	Bs	t_1_	t_2_	t_3_	Bs	t_1_	t_2_	t_3_
A	1	3.76	3.81	5.11	8.74	43.4	46.0	90.4	95.7	2.5	2.54	4.34	7.16
	2		4.16	4.52	7.76		43.9	52.7	81.0		3.74	6.46	22.6
	3		3.85	4.13	4.25		37.5	43.7	43.8		7.24	16.1	28.1
	4		4.16	5.17	5.79		46.8	60.5	79.9		3.09	7.13	26.8
B	1	9.61	10.8	15.2	16.3	40.3	68.3	115	131	10.3	10.5	16.1	17.4
	2		17.8	41.6	79.7		381	743	971		40.6	68.2	85.2
	3		9.68	9.70	13,4		41.6	42.3	62.1		53.2	838	4071
	4		11.2	15.1	17.7		57.7	130	132		12.5	22.1	31.6
C	1	2.75	2.86	4.19	7.50	19.2	28.3	49.3	127	<1	<1	1.40	2.08
	2		2.93	5.21	9.93		31.0	51.9	96.7		3.20	6.43	6.94
	3		2.76	3.71	4.72		19.7	19.8	30.6		4.38	24.4	121
	4		3.39	5.60	6.11		31.5	39.0	56.9		<1	1.84	2.84
D	1	17.3	17.6	30.1	31.4	290	293	341	396	6.1	6.20	15.4	17.6
	2		19.6	29.6	29.8		311	316	334		10.4	18.4	20.4
	3		18.3	29.7	29.9		292	302	310		15.4	113	156
	4		17.4	25.3	28.1		298	321	325		6.29	13.1	14.4
E	1	7.04	7.25	12.1	12.3	25.1	166	136	71	4.13	4.44	8.87	9.19
	2		7.10	28.2	40.8		163	541	705		8.60	61.9	63.14
	3		7.56	7.61	7.84		29	26	34.7		117	1061	3772
	4		8.24	11.5	12.3		47	94	95.1		4.94	9.02	36.7
H	1	<1	<1	<1	1.24	3.04	9.15	10.5	18.6	<1	<1	<1	<1
	2		<1	<1	9.27		10.4	13.7	69.4		1.82	2.90	13.1
	3		<1	<1	1.29		9.54	11.30	19.9		4.86	17	146
	4		<1	1.27	1.60		12.1	33.2	44.1		<1	<1	<1
		**Cr µg/kg**				**Fe µg/kg**				**Ni µg/kg**			
		Bs	t_1_	t_2_	t_3_	Bs	t_1_	t_2_	t_3_	Bs	t_1_	t_2_	t_3_
F	1	99	120	183	185	458	607	653	1100	437	464	1325	1605
	2		205	220	272		636	1878	1901		567	2105	3675
	3		174	189	235		570	853	1045		1748	40,564	143,472
	4		127	173	189		201	405	744		1355	1348	1726
G	1	11.2	50.3	56	342	46.4	85	114	2213	<12	<12	<12	198
	2		11.8	12.4	1988		534	7644	15,493		17.9	699	1806.9
	3		21.3	91.3	<10		49.4	238	64.2		8707	41,193	96,372
	4		24.2	342	440		122	974	3740		<12	901	382
